# On the Biliary Secretion of the Dog with Reference to the Action of Cholagogues

**Published:** 1879-05

**Authors:** 


### On the Biliary Secretion of the Dog with Reference to the Action of Cholagogues.

Dr. Will Rutherford, Professor of the Institutes of Medicine in the University of Edinburgh, has presented a report on this subject to the Scientific Grants Committee of the British Medical Association (*Brit. Med. Journ.*, Feb. 8, 1879), of which the following is the summary of result obtained.

1. In a curarized dog that has fasted eighteen hours, the secretion of bile is tolerably uniform during the first four or five hours after the commencement of the experiment, but falls slightly as a longer period elapses. Its composition remains constant.

2. Croton-oil is a hepatic stimulant of very feeble power. The high place assigned to it by Röhrig was probably the result of his imperfect method of experiment.

3. Podophyllin is a very powerful stimulant of the liver. During the increased secretion of bile, the percentage amount of the special bile solids is not diminished. If the dose be too large, the secretion of bile is not increased. It is a powerful intestinal irritant.

4. Aloes is a powerful hepatic stimulant. It renders the bile more watery, but at the same time increases the excretion of biliary matter by the liver.

5. Rhubarb is a certain, though not a powerful, hepatic stimulant. The bile secreted under its influence has the normal composition.

6. Senna is a hepatic stimulant of very feeble power. It renders the bile more watery.

7 Colchicum increases to a considerable extent the amount of biliary matter excreted by the liver, although it renders the bile more watery.

8. Taraxacum is a very feeble hepatic stimulant.

9. Scammony is a very feeble hepatic stimulant.

10. Gamboge is an intestinal, but not a hepatic, stimulant.

11. Castor-oil stimulates the intestinal glands, but not the liver.

12. Calomel stimulates the intestinal glands, but not the liver.

13. Euonymin is a powerful hepatic stimulant. It is not nearly so powerful an irritant of the intestine as podophyllin.

14. Sanguinarin is a powerful hepatic stimulant. It also stimulates the intestine, but not nearly so powerfully as podophyllin.

15. Iridin is a powerful hepatic stimulant. It also stimulates the intestine, but not so powerfully as podophyllin.

16. Leptandria is a hepatic stimulant of moderate power. It is a feeble intestinal stimulant.

17. Ipecacuan is a powerful hepatic stimulant. It increases slightly the secretion of intestinal mucus; but has no other stimulant effect on the intestine. The bile secreted under the influence of ipecacuan has the normal composition.

18. Colocynth is a powerful hepatic as well as intestinal stimulant. It renders the bile more watery, but increases the secretion of biliary matter.

19. Jalap is a powerful hepatic as well as intestinal stimulant.

20. Sodium-sulphate is a hepatic stimulant of considerable power. It also stimulates the intestinal glands.

21. Magnesium-sulphate is an intestinal but not a hepatic stimulant.

22. Potassium-sulphate is a hepatic and intestinal stimulant of considerable power. Its action on the liver is, however uncertain, probably owing to its sparing solubility.

23. Sodium-phosphate is a powerful hepatic, and a moderately powerful intestinal stimulant.

24. Rochelle salt is a feeble hepatic but a moderately powerful intestinal stimulant.

25. Ammonium-chloride stimulates the intestinal glands, but not the liver.

26. Dilute nitrohydrochloric acid is a hepatic stimulant of considerable power.

27. Mercuric chloride (corrosive sublimate) is a powerful hepatic stimulant, while it is a feeble intestinal stimulant. Although calomel is an intestinal but not a hepatic stimulant, excitement of the liver as well as of the intestinal glands results when mercuric chloride and calomel are administered together.

28. Calabar bean stimulates the liver, but not powerfully, unless it be given in very large doses.

29. Atropia-sulphate antagonizes the effect of Calabar bean on the liver, and thereby reduces the hypersecretion of bile produced by that substance. It does not, however, arrest the secretion of bile, and, when given alone, does not notably affect it.

30. Menispermin does not stimulate the liver. It slightly stimulates the intestinal glands.

31. Baptisin is a hepatic and also an intestinal stimulant of considerable power.

32. Phytolaccin is a hepatic stimulant of considerable power. It also slightly stimulates the intestinal glands.

33. Acetate of lead, in large doses, somewhat diminishes the secretion of bile, probably by a direct action on the liver.

34. Ammonium-phosphate is a moderately powerful hepatic stimulant of the liver. It does not stimulate the intestinal glands.

35. Tannic acid does not affect the secretion of bile.

36. Hydrastin is a moderately powerful hepatic stimulant, and a feeble intestinal stimulant.

37. Juglandin is a moderately powerful hepatic and a mild intestinal stimulant.

38. Sodium-benzoate is a powerful hepatic stimulant. It is not an intestinal stimulant.

39. Ammonia-benzoate stimulates the liver, but not quite so powerfully as the sodium-salt of benzoic acid. It does not stimulate the intestinal glands.

40. Benzoic acid stimulates the liver, but, owing to its insolubility, its action is less rapid and much less powerful than that of its alkaline salts.

41. Sodium salicylate is a very powerful hepatic stimulant. It does not notably stimulate the intestinal glands.

42. Sodium-chloride is a very feeble hepatic stimulant.

43. Sodium-bicarbonate has scarcely any appreciable effect as a hepatic stimulant, even when given in very large doses.

44. Potassium-bicarbonate freely excites the liver, and that only when given in very large doses.

45. Potassium-iodide has no notable effect on the biliary secretion.

46. Sulphate of manganese does not excite the liver, though it is a powerful excitant of the intestinal glands.

47. Morphia has no appreciable effect on the secretion of bile, and does not prevent the stimulating effect of such a substance, as sodium-salicylate.

48. Hyoscyamus does not notably affect the biliary secretion, and does not interfere with the stimulating effect of such a substance as sodium-salicylate.

49. Pure diluted alcohol does not affect the biliary secretion.

50. Jaborandi is a very feeble hepatic stimulant.

All the above conclusions are based on experiments performed on the dog, and have no reference to any observations made on the human subject.—*Amer. Jour. Med. Sciences.*

